# Promoting effect of a calcium-responsive self-assembly β-sheet peptide on collagen intrafibrillar mineralization

**DOI:** 10.1093/rb/rbac059

**Published:** 2022-09-05

**Authors:** Zhongcheng Li, Qian Ren, Sili Han, Longjiang Ding, Xi Qin, Die Hu, Ting He, Tian Tian, Ziqian Lu, Linglin Zhang

**Affiliations:** State Key Laboratory of Oral Diseases, National Clinical Research Centre for Oral Diseases, Department of Cariology and Endodontics, West China Hospital of Stomatology, Sichuan University, Chengdu 610041, Sichuan, China; State Key Laboratory of Oral Diseases, National Clinical Research Centre for Oral Diseases, Department of Cariology and Endodontics, West China Hospital of Stomatology, Sichuan University, Chengdu 610041, Sichuan, China; State Key Laboratory of Oral Diseases, National Clinical Research Centre for Oral Diseases, Department of Cariology and Endodontics, West China Hospital of Stomatology, Sichuan University, Chengdu 610041, Sichuan, China; State Key Laboratory of Oral Diseases, National Clinical Research Centre for Oral Diseases, Department of Cariology and Endodontics, West China Hospital of Stomatology, Sichuan University, Chengdu 610041, Sichuan, China; Department of Oral Medicine, Shenzhen Stomatology Hospital, Shenzhen 518038, Guangdong, China; State Key Laboratory of Oral Diseases, National Clinical Research Centre for Oral Diseases, Department of Cariology and Endodontics, West China Hospital of Stomatology, Sichuan University, Chengdu 610041, Sichuan, China; State Key Laboratory of Oral Diseases, National Clinical Research Centre for Oral Diseases, Department of Cariology and Endodontics, West China Hospital of Stomatology, Sichuan University, Chengdu 610041, Sichuan, China; State Key Laboratory of Oral Diseases, National Clinical Research Centre for Oral Diseases, Department of Cariology and Endodontics, West China Hospital of Stomatology, Sichuan University, Chengdu 610041, Sichuan, China; State Key Laboratory of Oral Diseases, National Clinical Research Centre for Oral Diseases, Department of Cariology and Endodontics, West China Hospital of Stomatology, Sichuan University, Chengdu 610041, Sichuan, China; State Key Laboratory of Oral Diseases, National Clinical Research Centre for Oral Diseases, Department of Cariology and Endodontics, West China Hospital of Stomatology, Sichuan University, Chengdu 610041, Sichuan, China

**Keywords:** collagen, polyacrylic acid, biomineralization, biomimetic materials, amorphous calcium phosphate

## Abstract

Recently, a *de novo* synthetic calcium-responsive self-assembly β-sheet peptide ID8 (Ile-Asp-Ile-Asp-Ile-Asp-Ile-Asp) has been developed to serve as the template inducing hydroxyapatite nucleation. The aim of this study was to evaluate the effect of ID8 on intrafibrillar mineralization of collagen making full use of its self-assembly ability. The mineralization experiments were carried out *in vitro* on both bare Type I collagen and fully demineralized dentin samples. The calcium-responsive self-assembly of ID8 was revealed by circular dichroism spectrum, 8-anilino-1-naphthalenesulfonic acid ammonium salt hydrate assay, attenuated total reflection Fourier transform infrared spectrum (ATR-FTIR) and transmission electron microscope (TEM). Polyacrylic acid (450 kDa) with a concentration of 100 μg ml^−1^ was selected as the nucleation inhibitor based on the determination of turbidimetry and TEM with selected area electron diffraction (TEM-SAED). The results showed that collagen intrafibrillar mineralization was significantly promoted with the pretreatment of self-assembly ID8 detected by TEM-SAED, SEM, X-ray diffraction and ATR-FTIR. The pretreatment of collagen utilizing self-assembly ID8 not only enhanced intermolecular hydrogen bonding but also contributed to calcium retention inside collagen and significantly increased the hydrophilicity of collagen. These results indicated that peptides with self-assembly properties like ID8 are expected to be potential tools for biomimetic mineralization of collagen.

## Graphical Abstract

**Figure:**
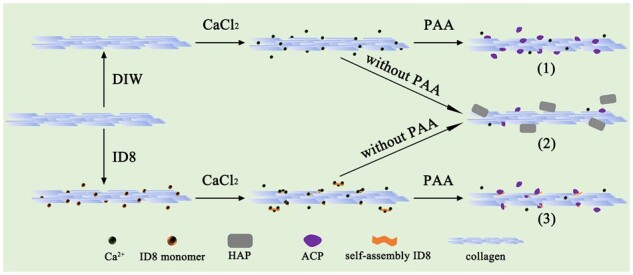

## Introduction

Dentin is a hierarchical hybrid material with excellent mechanical properties. More than 90% of the organic component in dentin is Type I collagen, which provides the structural framework for apatite deposition and facilitates to enhance dentin mechanical properties [1–3]. The mineral phase in dentin can be divided into intrafibrillar mineral and extrafibrillar mineral, in which the former is the major contributor to the mechanical properties of dentin [4–6]. Furthermore, intrafibrillar minerals have a clear protective effect on collagen structure [7–9]. As a result, collagen intrafibrillar mineralization has attracted the attention of dental clinicians for years.

Abundant significant work has been made toward intrafibrillar mineralization of collagen and was well reviewed [3, 10, 11]. From the conventional ion-based top-down crystallization concept to the bottom-up nonclassical crystallization pathway mediated by amorphous calcium phosphate (ACP), a series of models had been established to explain the potential mechanisms involved in intrafibrillar mineralization, such as the classical precipitation and growth of apatite [10, 12], polymer-induced liquid-precursor (PILP) process [13], mineralization by inhibitor exclusion [14], Columbic attraction [15] and Gibbs–Donnan equilibrium [16]. Most recently, some new strategies have been applied for collagen mineralization, such as synchronous self-assembly/mineralization of collagen [17], fluid shear stress-mediated collagen intrafibrillar mineralization [18] and collagen mineralization induced by polyelectrolyte–calcium complexes [19]. In fact, benefit from the identification of transient mineral phases (e.g. ACP) as the precursor and the development of PILP hypothesis, intrafibrillar mineralization of collagen has been well established through many strategies. Meanwhile, most theories also attempted to combine calcium and phosphorus in the form of ACP prior to mineralization, rather than investigating them as separate mineralization media. In this sense, the success of polyelectrolyte–calcium complexes on collagen intrafibrillar mineralization built a bridge between the classical and nonclassical pathways and demonstrated the possibility that calcium and phosphorus act as the separate medium to induce mineralization [19].

Hierarchical collagen mineralization is highly controlled by noncollagenous proteins (NCPs) *in vivo*. Self-assembly β-sheet conformation in NCPs was supposed to play a pivotal role during biomineral formation. For instance, dentin matrix protein 1 (DMP-1) is responsible for regulation of crystal nucleation and growth during dentinogenesis [20]. *In vitro* studies verified that the intermolecular assembly of functional domains into a β-sheet template was essential for the observed mineral nucleation induced by DMP-1 and there was a lattice-match effect between β-sheet conformation and hydroxyapatite (HAP) [21–23]. Furthermore, synthetic peptides with self-assembly β-sheet conformation such as P_11_-4 and P26 also showed active regulatory roles on crystal nucleation and collagen mineralization [24–26]. Inspired by these works, a *de novo* synthetic calcium-responsive self-assembly β-sheet peptide ID8 (Ile-Asp-Ile-Asp-Ile-Asp-Ile-Asp) was designed based on ionic self-complementarity in our previous work. ID8 was designed rationally based on the (XZXZ)_n_ sequence template with shorter and optimized sequence patterns, where X represented nonpolar amino acid, Z represented polar amino acid and n represented the number of repeats. Our previous studies showed that ID8 could serve as the template to induce HAP nucleation and promote biomimetic mineralization of initial caries lesions [27]. The self-assembly of ID8 can chelate large amounts of calcium and serve as the polyelectrolyte–calcium complexes. It is speculated that ID8 may be a potential tool regulating collagen intrafibrillar mineralization.

Therefore, the aim of this study is to investigate the effect of ID8 pretreatment on collagen properties and evaluate the polyelectrolyte–calcium complexes formed by self-assembly of ID8 on collagen intrafibrillar mineralization, which will also explore the potential of peptides with self-assembly properties as new tools for collagen mineralization.

## Materials and methods

### Chemicals

The peptide ID8 was synthesized, identified and purified to 95% purity through solid-phase synthesis by GL Biochem (Shanghai, China). Type I collagen derived from rat tails was obtained commercially from Sigma Aldrich (C7661, USA). Polyacrylic acid (PAA, 450 kDa), PBS powder and phosphoric acid were purchased from Macklin (Shanghai, China); 8-anilino-1-naphthalenesulfonic acid ammonium salt hydrate (1,8-ANS) was purchased from ApexBio (USA). Dimethylformamide (DMF) was purchased from Aladdin (Shanghai, China). All other chemicals were purchased from J&K Scientific (Beijing, China). Specially, the ID8 solution (pH 7.4) was prepared freshly when used by dissolving the peptide powder in deionized water (DIW) at room temperature.

### Characterizations of peptide ID8

#### Zeta potential

The ID8 solution (1 μg ml^-^^1^) with various pH values (3, 3.5, 4, 5, 7, 9, 11) were prepared using DIW, the zeta potentials of the solution were determined using a Zetasizer Nano ZS (Malvern) by the electrophoretic light scattering method. The conductivity range was 0–200 ms cm^−1^. To ensure repeatability, each group was repeated five times.

#### Circular dichroism

The ID8 solution (200 μM) for characterization was prepared in DIW in the absence and presence of calcium (1.5 mM CaCl_2_). The circular dichroism (CD) spectra were obtained over the range of 190–230 nm at room temperature using a CD spectropolarimeter (Chirascan, Applied Photophysics, UK). All spectra were obtained using a 2-nm bandwidth and a 1 nm s^−1^ scanning rate and were normalized to mean residual ellipticity values.

#### 1,8-ANS assay

The ID8 solution (200 μM) for characterization was prepared in DIW in the absence and presence of calcium (1.5 mM CaCl_2_). The 1,8-ANS solution (20 μM) was prepared freshly using DMF. The samples (1 ml) were mixed with ANS solution (1 μl) under dark conditions. After being incubated for 15 min, the samples were transferred to a 96-well plate at 250 μl per well, DIW was used as the control. The fluorescence measurements were conducted on a Fluorescence spectrometer F-2500 (Hitachi High-Technologies Corporation, Tokyo, Japan) with the excitation at 369 nm (6-nm bandwidth), and the fluorescence emission was collected at 450–550 nm (6-nm bandwidth). Each sample was measured five times in each measurement.

#### Attenuated total reflection Fourier transform infrared spectrum

The self-assembly ID8 solution (200 μM) was prepared using DIW with 1.5 mM CaCl_2_. After incubation for 15 min at room temperature, the solution was frozen at −80°C and lyophilized in vacuum to obtain self-assembly ID8 powder. The original ID8 powder and the self-assembly ID8 powder were detected by attenuated total reflection Fourier transform infrared spectrum (ATR-FTIR) (Invenior, Bruker) with spectra recorded in the range of 400–4000 cm^−1^ wavenumber with a resolution of 4 cm^−1^ intervals using 16 scans. All of the spectrum were normalized at the amide I band for further comparison; and in the spectrum of self-assembly ID8 powder, the FTIR deconvolution of the amide I band (1600–1700 cm^−1^) was fitted and calculated. Omnic8.0 (Thermo Fisher Scientific Inc., MA, USA) and Peakfit4.12 software (SPSS Inc., Chicago, IL, USA) were used for baseline correction, smoothing, Fourier self-deconvolution and the second-order derivative fitting were used to quantitatively analyze the second-order derivative of Fourier transform infrared spectra.

#### Transmission electron microscope

The self-assembly ID8 powder was dispersed with anhydrous ethanol and dropped onto nickel mesh. After 1 min of staining with uranyl acetate (1%), the samples were detected by transmission electron microscope (TEM-2100 Plus Electron Microscope, Japan) detection at 200 keV.

### Concentration screening of PAA

#### Preparation of mineralization medium containing different concentrations of PAA

The mineralization medium was prepared adopting the method from Deshpande and Beniash to minimize the pH change during mineralization and guarantee the reproducibility of the results [28]. Briefly, a 10× PBS buffer (pH 7.7) was prepared using commercial PBS powder at first. Subsequently, phosphorus stock solution, calcium stock solution and PAA stock solution were prepared. In detail, the phosphorus stock solution was prepared by diluting 85 ml 10× PBS buffer to 500 ml with DIW at first and then adding 0.14196 mg Na_2_HPO_4_ powder. The calcium stock solution (6.68 mM) was prepared by dissolving 370.6732 mg CaCl_2_ powder in 500 ml DIW. The PAA stock solution with different concentrations (50, 100, 200, 400 and 800 μg ml^−1^) were prepared by dissolving corresponding amount of PAA powder in 500 ml DIW. Finally, the mineralization medium containing different concentrations of PAA were prepared by mixing equal volume of calcium stock solution and PAA stock solution (or DIW) at first, and then adding phosphorus stock solution to reach the final volume ratio as calcium stock solution:PAA stock solution (or DIW):phosphorus stock solution = 1:1:2. The ultimate mineralization medium contained 1.67 mM CaCl_2_, 1 mM Na_2_HPO_4_, 0.85× PBS (8.5 mM phosphate, 131.7 mM NaCl) and different concentrations of PAA (0, 12.5, 25, 50, 100 and 200 μg ml^−1^).

#### Turbidimetry

The turbidimetry detection was conducted inside 96-well plates. In detail, 50 μl calcium stock solution and 50 μl PAA stock solution (or DIW) with different concentration were mixed inside each well at first, and then phosphorus stock solution was seeded at 100 μl per well using a multichannel pipette. Finally, 50 μl sterile mineral oil was added into each well quickly using a multichannel pipette to prevent evaporation. Each concentration had three parallel control wells. After mixing, the 96-well plate was immediately transferred into an automatic multiskan spectrum (Biotek Eon, USA). The absorbance of each well was measured at 550 nm after incubated at 37°C for 5 min first and subsequent automatic measurements were performed every 10 min for 9 h totally; the plate was vibrated automatically before each measurement. Afterwards, the 96-well plate was transferred into an incubator at 37°C and measured after 1, 2, 3, 4, 5 and 7 days of incubation, respectively. This experiment was repeated three times.

#### TEM with selected area electron diffraction

The mineralization medium in the presence or absence of screened concentration of PAA after different time of incubation (5 min, 24 h and 7 days) was dropped on nickel grids and detected by TEM with selected area electron diffraction (TEM-SAED) (TEM-2100 Plus Electron Microscope, Japan) at 200 keV.

### Preparation of reconstituted collagen and collagen hydrogels

Collagen stock solution (3 mg ml^−1^) was prepared by dissolving commercial Type I collagen in 0.1 M acetic acid solution and stored at 4°C. Glycine buffer (pH = 9.2) containing 50 mM glycine and 200 mM KCl was prepared freshly for collagen reconstitution. In detail, reconstituted collagen solution (50 μg ml^−1^) was prepared by adding 16.7 μl collagen stock solution into 983.3 μl glycine buffer, vibrated immediately and incubated for 20 min at room temperature. The reconstituted collagen solution after incubation showed slight gelation without fiber precipitation. After that, the nickel grids were placed face up on wax slices at the bottom of a six-well plate, and 14 μl reconstituted collagen solution was dripped onto each nickel grid. Then DIW was added to the remaining wells of the plate to maintain a humid environment. The plate was sealed with Parafilm and placed in a 37°C incubator for 24 h. After incubation, the nickel grids were washed with DIW and up-floated face down on glutaraldehyde droplets (0.5 wt%, 200 μl) for 2 h, washed with DIW again and air-dried for further treatment and characterization.

Collagen hydrogels were prepared using the method of Liu *et al*. [29]. Briefly, 150 μl of Type I collagen stock solution was neutralized with ammonia vapor for 4 h. The collagen was left to gel by incubation at a constant temperature of 37°C for 10 h. Then the collagen hydrogels were washed with DIW until the pH remained neutral and freeze-dried.

### Pretreatment, characterization and mineralization of reconstituted collagen

#### Pretreatment of reconstituted collagen

Corresponding to subsequent mineralization experimental grouping, the collagen-coated nickel grids were randomly divided into four groups, including DIW group, PAA group, self-assembly ID8 group and ID8 monomer group. For DIW group, PAA group and self-assembly ID8 group, each nickel grid was first up-floated face down on a 200-μl droplet of DIW (DIW group and PAA group) or 400 μM ID8 solution (self-assembly ID8 group) for 15 min, after then 200 μl of calcium solution (3 mM) was added into the droplets and incubated for another 15 min (to induce the calcium-responsive self-assembly of ID8). For ID8 monomer group, the nickel grids were first up-floated face down on a 200-μl droplet of 200 μM ID8 solution for 15 min, washed with DIW and then the grids were up-floated face down on a 200-μl droplet of calcium solution (1.5 mM) for 15 min. Finally, all the grids were washed with DIW.

#### Attenuated total reflection Fourier transform infrared spectrum

The pretreated collagen was detected by ATR-FTIR (Invenior, Bruker) with spectra recorded in the range of 400–4000 cm^−1^ wavenumber with a resolution of 4 cm^−1^ intervals using 16 scans. All the spectrum were normalized at the amide I band for further comparison.

#### X-ray photoelectron spectroscopy

The pretreated collagen was detected by X-ray photoelectron spectroscopy (XPS) (AXIS Ultra DLD, Kratos) with Al Kα X-ray radiation operating at 10 kV and 7 mA for full spectrum and at 10 kV and 15 mA for partial spectrum.

#### Absorbance measurement of calcium

The collagen gels were randomly divided into three groups (*n *=* *3), including DIW group, self-assembly ID8 group and ID8 monomer group. For DIW group and self-assembly ID8 group, each collagen gel was first immersed in 2 ml DIW (DIW group) or 2 ml of 400 μM ID8 solution (self-assembly ID8 group) for 15 min, after then 2 ml of calcium solution (3 mM) was added and incubated for another 15 min. For ID8 monomer group, the collagen gel was first immersed in 2 ml ID8 solution (200 μM) for 15 min, washed with DIW and then the collagen gel was immersed in 2 ml of calcium solution (1.5 mM) for 15 min. The supernatant containing remaining calcium was filtered through 0.22-μm Millipore films. The concentrations of calcium (C_supernatant_) were determined using calcium kit (Nanjing Jiancheng Bioengineering). The principle of the kit is that calcium ions can form a blue complex with methylthymol blue in an alkaline environment. The concentration of calcium ions in the supernatant can be calculated by colorimetric comparison with the calcium ion standard liquid. The sensitivity of the kit is 0.005 mM and the linear range is 0–2 mM. Briefly, the supernatant and standard liquid were seeded at 10 μl per well in 96-well plate and reacted with 250 μl working solution, the absorbance of the solution was measured at 610 nm after being incubated at room temperature for 5 min. The contents of Ca^2+^ in collagen gel were expressed as 1.5 mM-C_supernatant_.

#### SEM with energy dispersive spectrum

The pretreated collagen was sputter coated with gold for 30 s and observed by SEM (Apreo S, FEI, Eindhoven, The Netherlands) at an accelerating voltage of 15 keV, and the energy dispersive spectrum (EDS) was obtained by the attached energy disperse spectroscopy (Aztec X-Max80).

#### Water contact angle measurement

The pretreated collagen gels were air-dried and the surface wetting behaviors were assessed by a contact angle meter (DSA25E, KRUSS, Germany) with sessile drop method at room temperature. Water droplets with the volume of 3 µl were carefully dropped onto the surfaces in three different positions per sample to obtain the average static contact angle value within 10 s.

#### Mineralization of reconstituted collagen

The pretreated collagen of DIW group was mineralized in the mineralization medium without PAA and that of PAA group, self-assembly ID8 group and ID8 monomer group were mineralized in the mineralization medium containing screened concentration of PAA as described above for 1 and 3 days. The mineralized collagen was detected by TEM-SAED (TEM-2100 Plus Electron Microscope, Japan) at 200 keV.

### Preparation of fully demineralized dentin samples

Extracted human third molars free of caries were collected with the patient’s approval. Ethical approval (WCHSIRB-D-2019-027) for the use of extracted human teeth was obtained in accordance with guidelines from the West China Hospital of Stomatology, Sichuan University.

Dentin samples with 2 ± 0.2 mm thickness were cut from the mid-coronal region of the selected teeth perpendicular to the tubule direction using a diamond blade with continuous water cooling (Struers Minitom, Struers, Copenhagen, Denmark). The sample surfaces were polished sequentially using water-cooled waterproof silicon carbide paper (1000, 1500, 2000, 3000 and 5000 grit). Fully demineralized dentin samples were obtained by demineralizing the dentin samples using 37 wt.% phosphoric acid for 2 days at 37°C and rinsed in DIW for 30 min with ultrasonic. The demineralized samples were randomly divided into three groups (*n *=* *15), including DIW group, PAA group and ID8 group.

### Pretreatment and mineralization of dentin samples

#### Pretreatment of dentin samples

The samples were first immersed in 2 ml of DIW (DIW group and PAA group) or ID8 solution (400 μM, ID8 group) for 15 min, after then 2 ml of calcium solution (3 mM) was added and incubated for another 15 min. Finally, the samples were washed with DIW for further mineralization.

#### Mineralization of dentin samples

The pretreated samples of DIW group were mineralized in the mineralization medium without PAA and that of PAA group and ID8 group were mineralized in the mineralization medium containing screened concentration of PAA. The mineralization medium was renewed daily. In detail, each dentin sample was mineralized in 4 ml of mineralization medium in 12-well plates. The plates were sealed with Parafilm and stirred at 100 rpm at 37°C for 3 and 7 days. At Day 3, all of the samples were cut in the middle. One half of each sample was put back into the mineralization medium for continuous mineralization till Day 7, and the other half was rinsed in DIW for 30 min with ultrasonic. The air-dried mineralized samples, representative fully demineralized samples and sound samples were immersed in 50%, 75% and 100% ethanol for gradient dehydration, 15 min at each concentration. Finally, the dehydrated samples were immersed in hexamethyldisilane for 1 h and air-dried for further characterization. At Day 7, the remaining samples were collected and treated with the same strategy for further characterization.

#### SEM

Dehydrated sound dentin samples, fully demineralized dentin samples and mineralized dentin samples were sputter coated with gold for 30 s and then observed by SEM (Inspect F, FEI, Eindhoven, The Netherlands) at an accelerating voltage of 20 keV.

#### Attenuated total reflection Fourier transform infrared spectrum

Dehydrated sound dentin samples, fully demineralized dentin samples and mineralized dentin samples were detected by ATR-FTIR (Invenior, Bruker) with spectra recorded in the range of 400–4000 cm^−1^ wavenumber with a resolution of 4 cm^−1^ intervals using 16 scans. The obtained infrared spectrum was normalized at the amide A peak for further comparison of mineral’s characteristic peaks.

#### X-ray diffraction

Dehydrated sound dentin samples, fully demineralized dentin samples and mineralized dentin samples were analyzed by X-ray diffraction (XRD) (EMPYREAN, The Netherlands) with Cu Kα radiation (*λ *= 1.54 Å) operating at 40 kV and 40 mA, a sampling step of 0.026 and a 2θ range of 10°–60°. Results were analyzed by MDI Jade 6.0 and compared with a standard HAP card (JCPDS 09-0432) to find corresponding peaks.

#### Transmission electron microscope with selected area electron diffraction

Collagen layers (∼0.5 mm) on the surfaces of dehydrated fully demineralized samples and mineralized samples were cut off with a blade and frozen in liquid nitrogen for 20 min. Afterwards, the collagen layers were ground into powders manually using mortar–pestle with liquid nitrogen. The powder was dissolved in ethanol and dispersed by ultrasonic. Finally, the dispersed powders were dripped onto the nickel mesh and air-dried for TEM-SAED observation (TEM-2100 Plus Electron Microscope, Japan) at 200 kV.

### Statistical analysis

SPSS 22.0 software (Chicago, IL, USA) was used for statistical analyses of data. All data were presented as mean ± SD. The comparison of the groups in absorbance measurement of calcium was performed by Student’s *t*-test and the comparison of the groups in water contact angle (WCA) measurement was performed by one-way ANOVA, followed by Tukey’s multiple comparisons test. *P *<* *0.05 was considered as statistically significant.

## Results

### Characterizations of peptide ID8

#### Zeta potential

**Figure 1.**
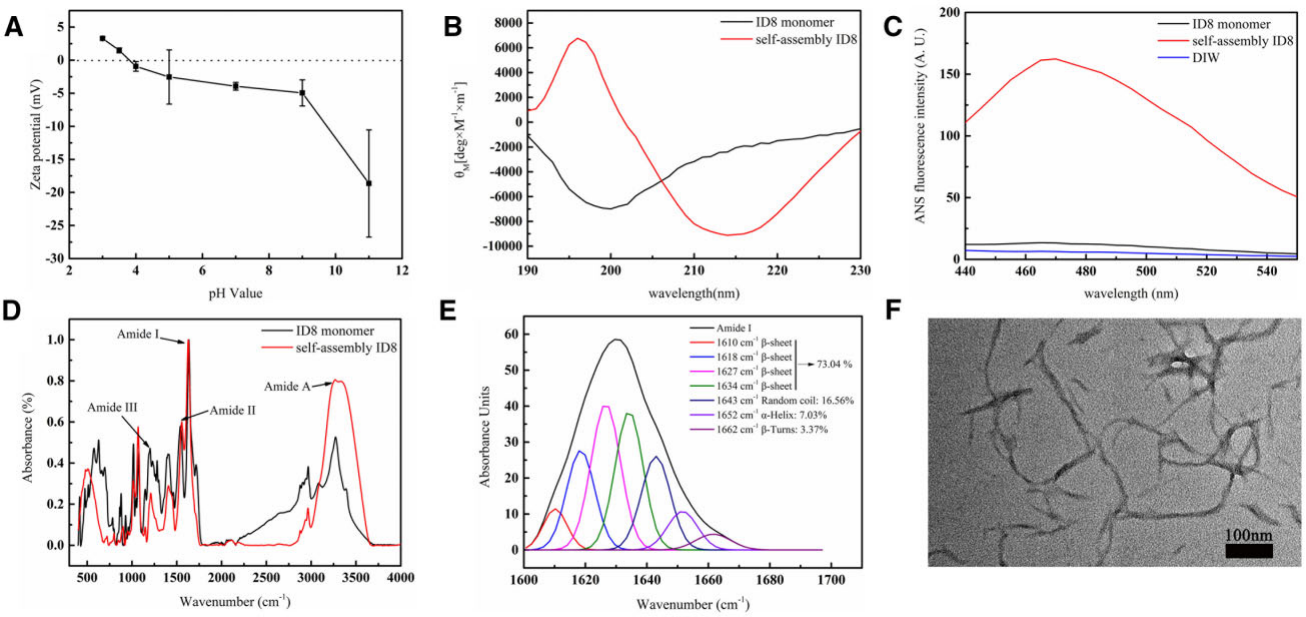
Characterizations of the peptide ID8. (**A**) Zeta potentials of ID8 solution at various pH values, reflecting the IP of ID8. (**B**) The normalized CD spectrum of ID8 solution with and without 1.5 mM calcium. (**C**) The fluorescence spectra of ID8 solution with and without 1.5 mM calcium in 1,8-ANS assay. (**D**) ATR-FTIR spectrum of self-assembly ID8 and ID8 monomer. (**E**) Curve-fitting analysis of ATR-FTIR spectra in amide I region of self-assembly ID8. (**F**) TEM image of self-assembly ID8.

As shown in Fig. 1A, the results demonstrated that the zeta potential of ID8 solution approached to zero between pH 3.5 and 4, indicating that the isoelectric point (IP) of ID8 was located between 3.5 and 4. The results also indicated that ID8 molecules carried net negative charges in a weak alkaline environment.

#### Circular dichroism

The calcium-responsive self-assembly of ID8 was explored by CD at physiological pH (Fig. 1B). The spectra of ID8 solution (200 μM) without calcium showed a characteristic random-coil conformation with a broad negative peak at 200 nm. Upon addition of calcium (1.5 mM) to ID8 solution, the spectra of ID8 displayed a characteristic β-sheet conformation with a strong positive peak at 196 nm and a strong negative peak at 214 nm.

#### 1,8-ANS assay

As shown in Fig. 1C, the ID8 solution in the absence of calcium showed weak fluorescence intensity, while the fluorescence intensity increased significantly with the addition of calcium, and the peak position also showed a slight blue shift from 470 to 465 nm.

#### Attenuated total reflection Fourier transform infrared spectrum

Compared with original ID8 powder, the ID8 powder obtained from ID8 solution in the presence of calcium showed significantly stronger peak at amide A band and a slight blue shift from 3272.7 to 3268.9 cm^−1^, indicating stronger intermolecular hydrogen bonding (Fig. 1D). Moreover, the fitting results of the amide I band (1600–1700 cm^−1^) of the self-assembly ID8 powder showed that the content of β-sheet structure in the secondary structure of self-assembly ID8 reached more than 73% (Fig. 1E).

#### Transmission electron microscope

As shown in Fig. 1F, self-assembly ID8 appeared as nanofibers in the presence of calcium under TEM observation.

### Concentration screening of PAA

#### Turbidimetry

**Figure 2.**
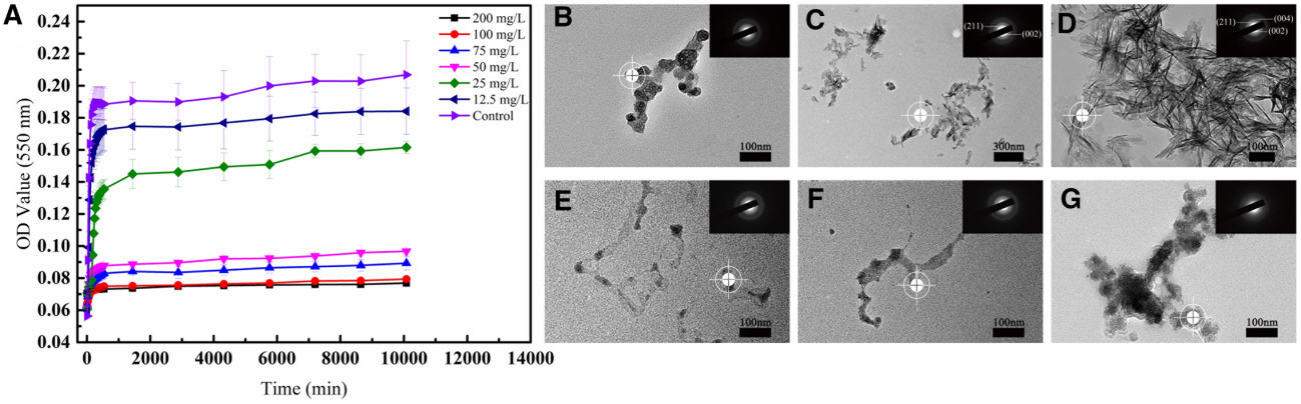
The nucleation inhibition of PAA with different concentrations. (**A**) Turbidimetry of mineralization medium containing different concentrations of PAA within 7 days. (**B**) The mineral phase in mineralization medium in the absence and presence of 100 μg ml^-1^ PAA under TEM-SAED within 7 days, the white targets marked the diffraction areas.

As shown in Fig. 2A, the absorbance increased rapidly over time at the initial stage of mineralization and reached a relatively constant maximum later. Compared with the control group (without PAA), the maximum absorbance of the experimental groups (with different concentrations of PAA) decreased to varying degrees. Nevertheless, when the PAA concentration reached 100 μg ml^−1^, the maximum absorbance almost no longer decreased.

#### Transmission electron microscope with selected area electron diffraction

As presented above, PAA with a concentration of 100 μg ml^−1^ was selected as the nucleation inhibitor. The minerals in the control group at 5 min were spherical ACP particles (Fig. 2B). These ACP particles crystallized into irregular plate crystals after 24 h (Fig. 2C) and grew into larger plate crystals after 7 days (Fig. 2D), as indicated by the narrow arc patterns at the 002 and 211 positions in SAED. Compared with the control group, the minerals in mineralization medium with 100 μg ml^−1^ PAA were ACP particles at 5 min (Fig. 2E) and there was no subsequent crystallization but fusion after 24 h (Fig. 2F) and 7 days (Fig. 2G) of incubation.

### Characterization of the pretreated collagen

#### Attenuated total reflection Fourier transform infrared spectrum

**Figure 3.**
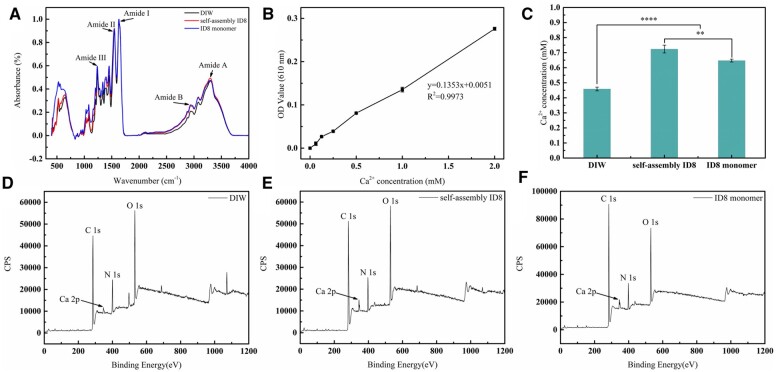
Characterizations of the pretreated collagen, revealing the interaction between ID8 and collagen. (**A**) ATR-FTIR spectrum of collagen with different pretreatment. (**B**) The linear relationship between calcium concentrations and absorbance at 610 nm. (**C**) The calcium absorption content in collagen gel with different pretreatment, data were presented as the mean ± SD. The statistical analysis was performed using Student’s *t*-test (**P <* *0.01, ****P <* *0.0001, *n *=* *3). (**D–F**) XPS full spectrum of the collagen with different pretreatment.

The pretreatment of collagen with DIW or ID8 did not alter the main structure of collagen, the characteristic amide I, II, III, A and B bands could still be found under different pretreatment. Compared with DIW pretreatment, collagen pretreated with ID8 showed a slight blue shift from 3306 to 3294 cm^−1^ (self-assembly ID8 group) and 3292 cm^−1^ (ID8 monomer group) at the amide A band, indicating stronger intermolecular hydrogen bonding (Fig. 3A).

#### Absorbance measurement of calcium

As shown in Fig. 3B, there was a good linear relationship between calcium concentration and absorbance at 610 nm (*R*^2^ = 0.9973). Compared with the DIW group (0.459 mM), the collagen gel in self-assembly ID8 group (0.724 mM) and ID8 monomer group (0.647 mM) showed significantly higher calcium absorption content in Fig. 2E (**P <* *0.01, ****P <* *0.0001, *n *=* *3).

#### X-ray photoelectron spectroscopy

The main elements on the collagen surface were carbon, nitrogen and oxygen. All spectrums expressed Ca 2p peaks, indicating a certain amount of Ca^2+^ on collagen (Fig. 3D–F). Semi quantitative analysis of the full spectrum showed that the relative content of Ca^2+^ on collagen treated with DIW was about 0.72 at%, while that on collagen treated with self-assembly ID8 increased significantly to 1.45 at% and that of ID8 monomer group increased to 1.24 at% (Table 1).

**Table 1.** Semi quantitative analysis of XPS full spectrum

	O 1s (at%)	N 1s (at%)	C 1s (at%)	Ca 2p (at%)
DIW	16.62	10.37	72.29	0.72
Self-assembly ID8	16.03	8.99	73.53	1.45
ID8 monomer	16.80	9.16	72.80	1.24

#### SEM with energy dispersive spectrum

**Figure 4.**
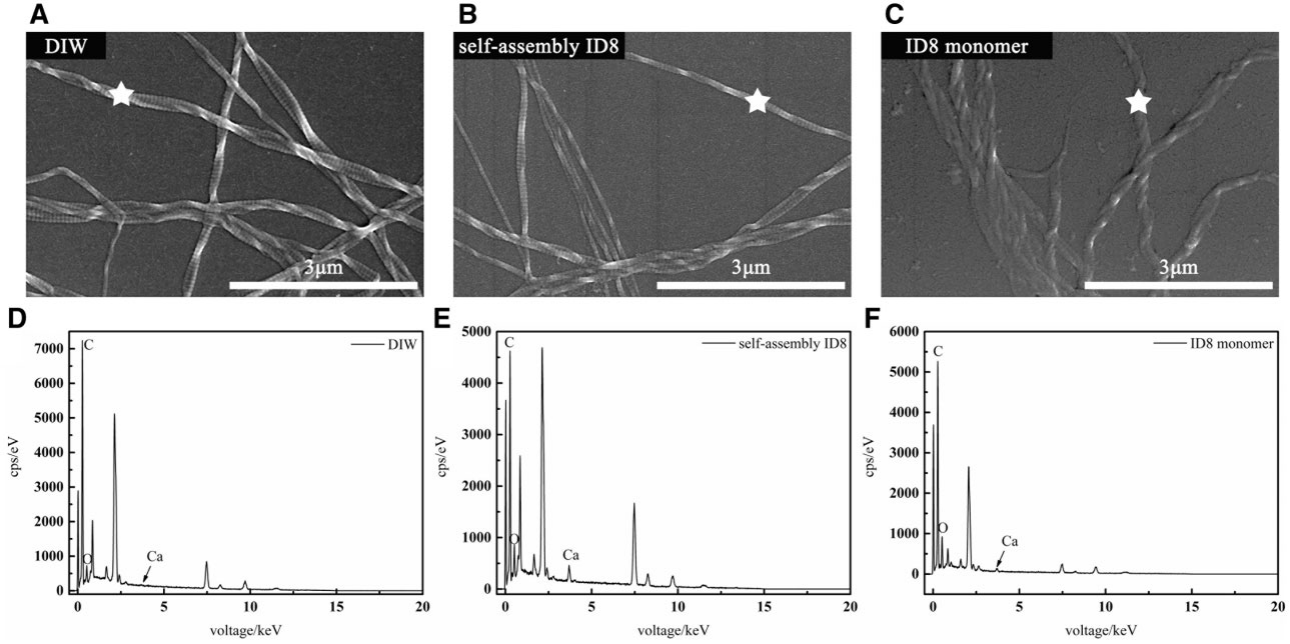
SEM-EDS of the pretreated collagen, exhibiting the calcium retention inside collagen of DIW group (**A**), self-assembly ID8 group (**B**) and ID8 monomer group (**C**). The corresponding EDS were displayed below and the white pentacles indicated the diffraction areas.

All pretreatments did not affect the characteristic periodic structure of collagen. The EDS spectrum (white pentacles indicated diffraction areas) of collagen pretreated with DIW showed weak Ca^2+^ peak, while that of collagen pretreated with self-assembly ID8 and ID8 monomer showed significantly stronger Ca^2+^ peaks (Fig. 4).

#### WCA measurement

**Figure 5.**
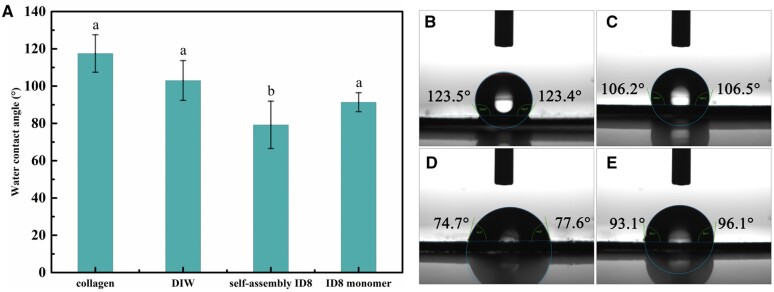
WCA of air-dried collagen gels with different pretreatment. (**A**) The WCA of different groups and data were presented as the mean ± SD. The statistical analysis was performed using one-way ANOVA, followed by Tukey’s multiple comparisons test (*P *<* *0.01, *n *=* *3). Representative WCA images of control group (**B**), DIW group (**C**), self-assembly group (**D**) and ID8 monomer group (**E**) were shown on the right.

As shown in Fig. 5, the air-dried collagen gel without pretreatment showed a mean static WCA of 117.51°, and the WCA decreased to 103.02° (DIW group) and 91.38 (ID8 monomer group). The collagen gel of self-assembly ID8 group showed much lower WCA than the other groups (79.25°).

### Mineralization of reconstituted collagen

#### Collagen mineralization of different groups

**Figure 6.**
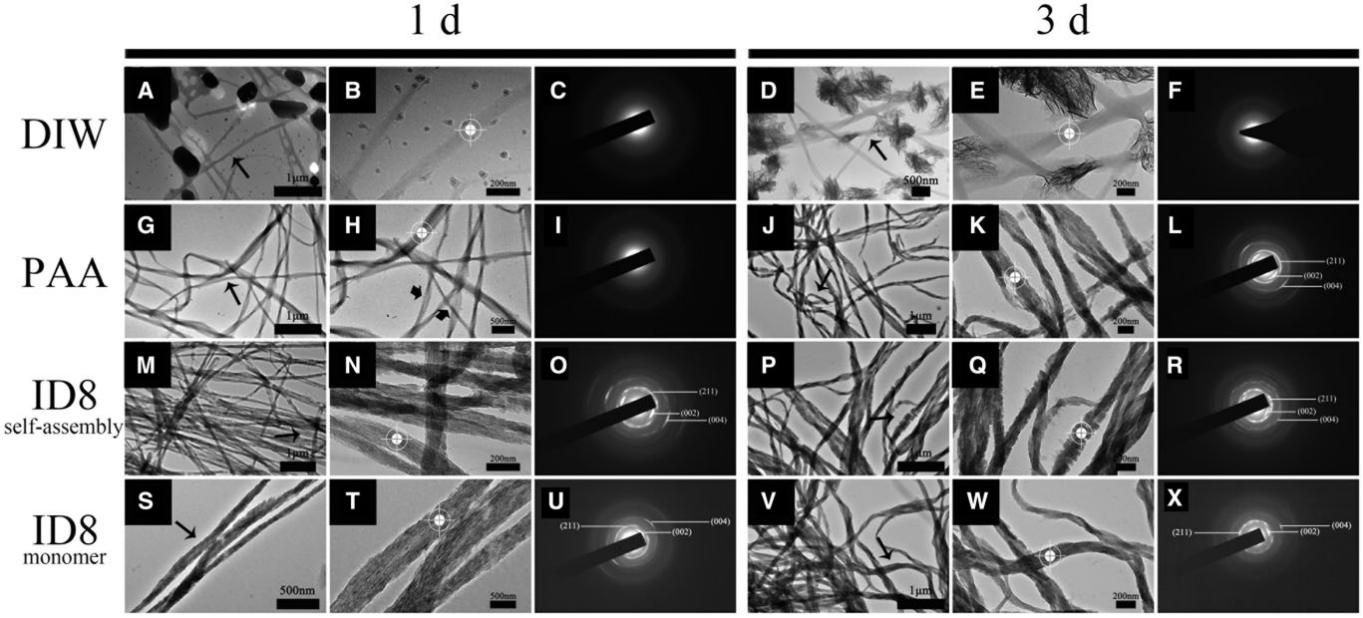
Collagen mineralization of different groups under TEM-SAED for 1 and 3 days. (**B, E, H, K, N, Q, T, W**) were corresponding zoom-in figures of the narrow black arrow-marked areas in (**A, D, G, J, M, P, S, V**), and the corresponding SAED patterns were shown in (**C, F, I, L, O, R, U, X**). The white targets marked the diffraction areas and the broad black arrows marked the periodic structure of unstained collagen.

As displayed in Fig. 6, collagen of DIW group exhibited no visible intrafibrillar mineralization at 1 day but many scattered plate crystals. These crystals grew and matured at 3 days and the collagen still showed no intrafibrillar mineralization. As the classical model, PAA group showed no scattered crystals, and the periodic structure of collagen was clearly visible without staining at 1 day, indicating ACP infiltration. Intrafibrillar mineralization was achieved at 3 days and the *c*-axis of the crystals was parallel to the collagen long axis, as indicated by the narrow arc patterns at 002 and 004 positions (white targets indicated diffraction areas). However, the collagen of self-assembly ID8 group and ID8 monomer group achieved severe intrafibrillar mineralization at 1 day and was further improved at 3 days, which also exhibited highly ordered orientation verified by the SAED. Specially, the mineralized collagen in self-assembly ID8 group still exhibited periodic structure, while that of PAA group at 3 days and ID8 monomer group at 1 day did not.

#### The mineralization process of collagen pretreated with self-assembly ID8

**Figure 7.**
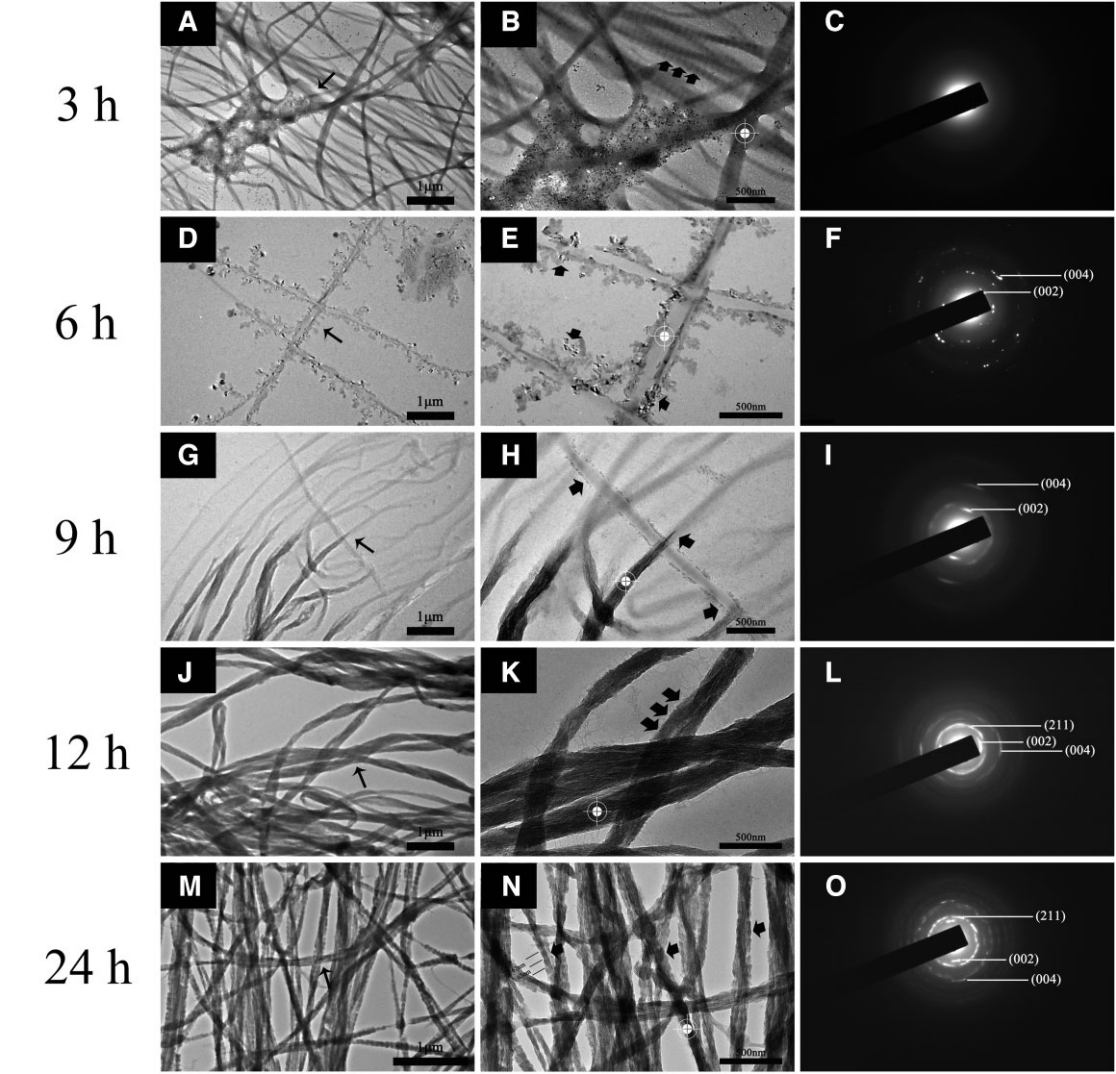
The promoted mineralization process of collagen in self-assembly ID8 group within 24 h under TEM-SAED. (**B, E, H, K, N**) were corresponding zoom-in figures of the narrow black arrow marked areas in (**A, D, G, J, M**), and the corresponding SAED patterns were shown in (**C, F, I, L, O**). The white targets marked the diffraction areas and the broad black arrows marked the broad black arrows marked the periodic structure, mineralization or crystals. As shown in (**N**), the mineralized collagen showed periodic pattern of about 67 nm.

Further studies explored the process of promoted mineralization in self-assembly ID8 group (Fig. 7). There were amorphous minerals in the field and the unstained collagen exhibited periodic structure at 3 h, indicating ACP infiltration. After 6 h of mineralization, there were abundant extrafibrillar minerals adsorbed on collagen, but the outline of collagen was still visible. The SAED on collagen showed diffraction patterns of scattered crystals, indicating the initial crystallization (white targets indicated diffraction areas). Partial collagen had realized intrafibrillar mineralization at 9 h, but the other areas were still in the infiltration process. Interestingly, the extrafibrillar minerals decreased significantly. The SAED on mineralized collagen showed slight arc patterns at 002 and 004 positions, indicating ordered orientation of crystals parallel to the collagen long axis, even if the poor crystallinity. Intrafibrillar mineralization was almost achieved at 12 h, and the crystals inside collagen exhibited highly ordered orientation verified by the SAED. After 24 h, intrafibrillar mineralization with periodic structure (as indicated by the black arrows) was achieved with higher crystallinity, as indicated by the smaller angles of arc patterns at 002 and 004 positions compared with that of 12 h.

### Mineralization of fully demineralized dentin samples

#### SEM

**Figure 8.**
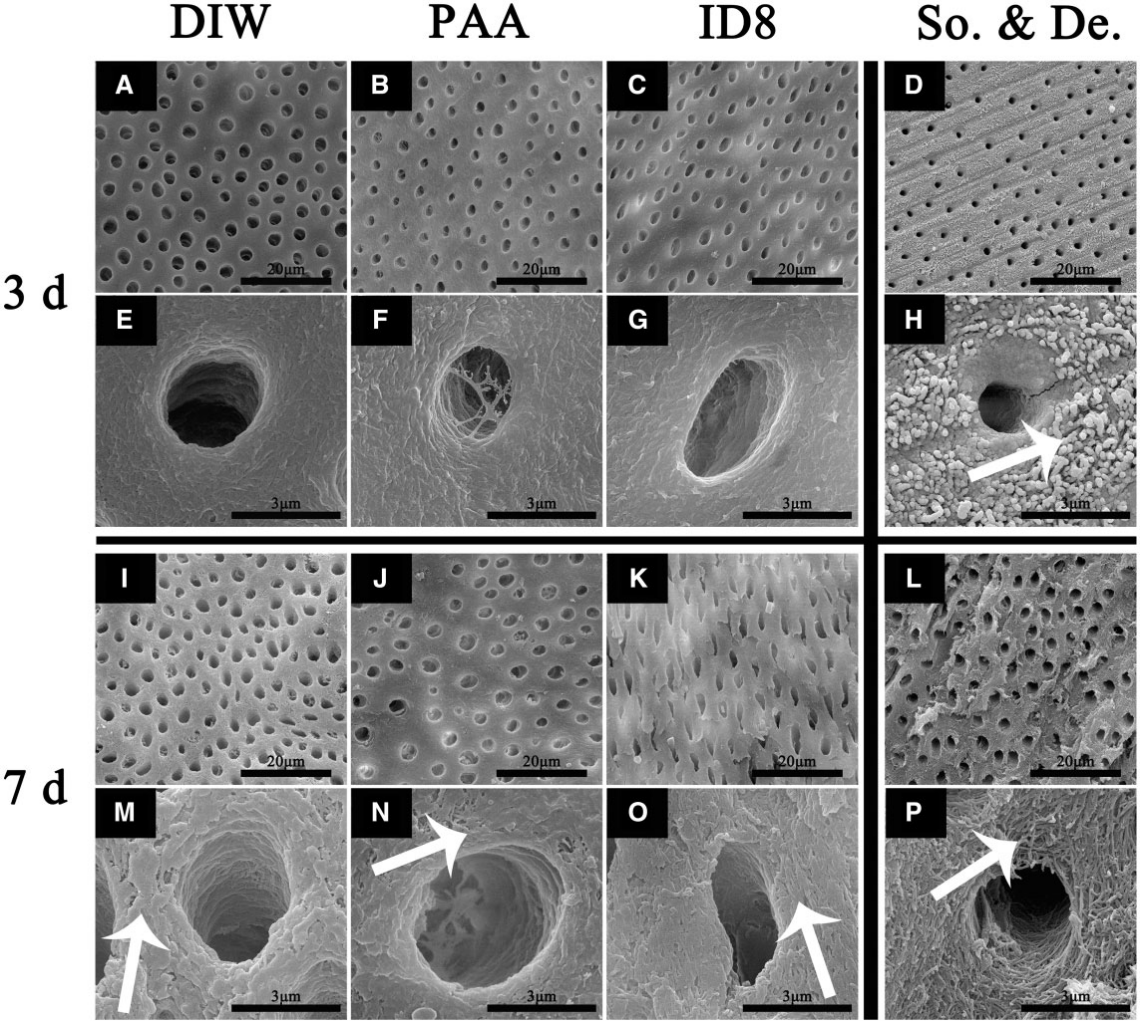
Mineralization of fully demineralized dentin samples under SEM for 3 and 7 days. (**E, F, G, H, M, N, O, P**) were corresponding zoom-in figures of (**A, B, C, D, I, J, K, L**), De. represents fully demineralized dentin samples and so. represents sound dentin samples. The white arrows indicated collagen fibrils wrapped by minerals, demineralized collagen fibrils, extrafibrillar crystals and collagen with visible periodic structure.

As shown in Fig. 8, the surface of sound dentin samples displayed a dense layer of minerals and there were some exposed collagen fibrils wrapped by minerals. After fully demineralization, the collagen fibrils on the sample’s surface and the inner wall of the tubules were exposed. After 3 days of mineralization, massive amorphous minerals deposited between collagen in all three groups. After 7 days of mineralization, there were abundant extrafibrillar crystals in DIW group and PAA group. Whereas, the self-assembly ID8 group exhibited much fewer extrafibrillar crystals and the collagen showed visible periodic structure (as indicated by the white arrow). All samples showed no occlusion of tubules during mineralization.

#### Attenuated total reflection Fourier transform infrared spectrum

**Figure 9.**
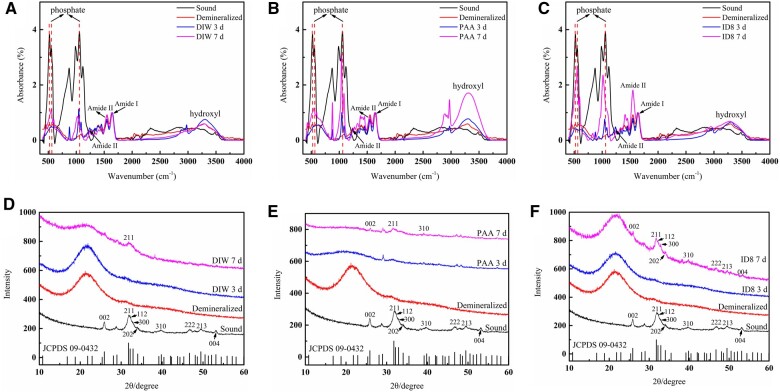
Mineralization of fully demineralized dentin samples under ATR-FTIR and XRD for 3 and 7 days. ATR-FTIR results of mineralized dentin samples in DIW group (**A**), PAA group (**B**) and self-assembly ID8 group (**C**) for 3 and 7 days. XRD results of mineralized dentin samples in DIW group (**D**), PAA group (**E**) and self-assembly ID8 group (**F**) for 3 and 7 days.

As shown in Fig. 9A–C, the sound dentin sample showed strong phosphate peaks at 523, 557 and 1057 cm^−1^. After demineralization, these peaks decreased significantly, indicating the complete demineralization of samples. After 3 days of mineralization, samples still displayed weak peaks at these positions in all three groups, indicating poor mineralization. After 7 days of mineralization, samples of the self-assembly ID8 group showed the strongest phosphate peaks compared with the other two groups and the single peak at ∼580 cm^−1^ had split into two peaks at 555 and 598 cm^−1^, implying good crystallinity.

#### X-ray diffraction

As shown in Fig. 9D–F, the sound dentin sample adopted a standard spectrum of carbonated HAP. After demineralization, the characteristic peaks of HAP disappeared, confirming the complete demineralization of samples. After 3 days of mineralization, samples exhibited no obvious peaks at 002, 211 and 004 positions in all three groups, indicating the poor crystallinity. After 7 days of mineralization, the samples in DIW group and PAA group displayed weak peaks at 002, 211 and 004 positions, while the samples in self-assembly ID8 group showed stronger peaks at 002, 211 and 004 positions.

#### Transmission electron microscope with selected area electron diffraction

**Figure 10.**
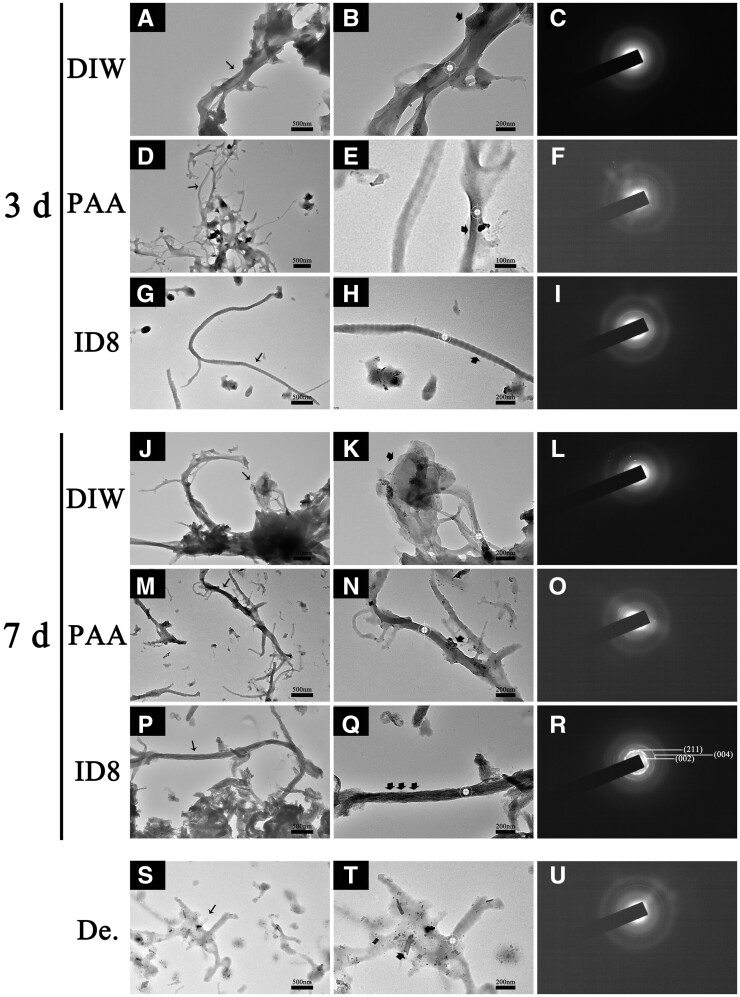
TEM-SAED of the collagen layers on the surfaces of dentin samples after mineralization for 3 and 7 days. (**B, E, H, K, N, Q, T**) were corresponding zoom-in figures of the narrow black arrow marked areas in (**A, D, G, J, M, P, S**), and the corresponding SAED patterns were shown in (**C, F , I , L , O, R, U**). De. represents fully demineralized dentin samples.

As shown in Fig. 10, the unstained collagen of fully demineralized sample showed low electron density, and there were still a few crystal residues between collagen but the periodical structure disappeared. After 3 days of mineralization, collagen of DIW group was wrapped by amorphous extrafibrillar minerals as indicated by the corresponding SAED. Collagen of PAA group showed a higher electron density than demineralized collagen, indicating ACP infiltration. Similarly, collagen of the self-assembly ID8 group exhibited periodical structure, indicating ACP infiltration. After 7 days of mineralization, collagen of DIW group showed many large extrafibrillar crystals around the collagen. In PAA group, the periodic structure appeared and the SAED on collagen indicated that the minerals inside collagen were mainly ACP. Collagen of self-assembly ID8 group showed severe hierarchical intrafibrillar mineralization and the crystals inside collagen exhibited ordered orientation parallel to the collagen long axis, as indicated by the arc 002 and 004 patterns.

## Discussion

In recent years, bioactive molecules adopted great success in controlling collagen intrafibrillar mineralization through precursor stabilization, mineralization promoting and mineralization regulating [30–34]. Generally, these biomolecules mainly involve bioactive small molecules (amino acids, citrate, pyrophosphate, etc.), bioactive macromolecules (proteins, peptides, poly(amido amine) dendrimers, etc.) and some polyelectrolytes (polycation, polyanion and amphoteric polyelectrolyte). Among them, peptides with self-assembly properties showed attractive prospects due to their potential substitution effect on NCPs [24, 35]. In this study, the effect of a calcium-responsive self-assembly β-sheet peptide ID8 on collagen intrafibrillar mineralization was explored.

Mineralization medium usually showed a profound impact on collagen mineralization. In order to guarantee the reproducibility of the experiment and reduce the impact of pH change, the mineralization medium was prepared utilizing PBS buffer as part of the phosphorus source [28]. In this study, PAA was selected as the nucleation inhibitor of ACP [36]. In biomineralization, two types of NCPs are generally considered as the key element, one is to stabilize ACP (sequestration motif) and the other is to initiate and regulate nucleation (templating motif). PAA are often employed as sequestration motif analogues to stabilize ACP [37]. Both the molecular weight (MW) and the concentration of PAA are correlated with PAA-ACP stability and growth in mineralization medium, and then affect the way of collagen mineralization. Either too strong or too weak PAA-ACP stability is not suitable for inducing collagen intrafibrillar mineralization. Anyhow, PAA is an ideal NCPs substitute for biomimetic mineralization of collagen and there is a wide range of combined MW and concentration of PAA that is suitable for intrafibrillar mineralization of collagen [36]. In addition, to avoid the dispute over whether nucleation inhibitor enters collagen during mineralization, PAA (450 kDa, ≫40 kDa) was selected based on collagen’s size exclusion characteristic [38]. The effective concentration of PAA for ACP stabilization was explored utilizing turbidimetry [39]. PAA showed concentration-dependent stabilization efficacy and the efficacy reached maximum when the concentration reached 100 μg ml^−1^. As a result, 100 μg ml^−1^ PAA was selected as the nucleation inhibitor that could stabilize ACP within 7 days under TEM-SAED.

The IP of ID8 was located in 3.5–4 as presented by zeta potential measurement, indicating that ID8 molecules carried net negative charges in weak alkaline environment. The negatively charged feature of ID8 made it easy to actively collect calcium from the solution and form polyelectrolyte–calcium complexes, and then play a regulatory role in mineralization. This made ID8 similar to many NCPs. Studies proved that NCPs involved in dentin and bone mineralization are usually acidic and negatively charged. Such negatively charged proteins/domains facilitate avid binding to Ca^2+^ and ${\text{PO}}_{4}^{3-}$ ions which are supersaturated in the ECM milieu, and then responsible for mineral nucleation, crystal growth, HAP crystallinity, inhibition of nonspecific mineral deposition and so on [11]. The calcium-responsive self-assembly of ID8 at experimental calcium concentration (1.5 mM) was confirmed by CD, 1,8-ANS assay and ATR-FTIR. The secondary structure of ID8 changed significantly with the addition of calcium, and finally adopted a typical β-sheet conformation under CD; 1,8-ANS is a hydrophobic fluorescent probe, which is widely used to detect the transformation of protein structure. ANS has a high affinity for the hydrophobic surface of the peptide, and its sulfonic group interacts with the cationic group of the peptide through electrostatic interaction to form a hydrophobic microenvironment for its own aromatic ring, making it emit fluorescence. Therefore, the significantly increased fluorescence intensity in self-assembly ID8 group indicated that ID8 self-assembled in the presence of calcium and formed a large number of hydrophobic centers. The content of β-sheet in the secondary structure of the self-assembly ID8 reached more than 73% under ATR-FTIR and the ID8 monomer molecules aggregate into nanofibers observed by TEM. The self-assembly secondary structures of peptides and proteins are usually critical to their biological functions. For example, the triple helix structure of collagen is the basis of its scaffold effect, the self-assembly β-sheet conformations are essential for DMP-1 and Type II TGF-β receptor interacting protein 1 to perform their biological functions [11, 22, 23, 40]. Therefore, ID8 had a conformational basis in regulating collagen mineralization. Moreover, ID8 self-assembly was calcium-dependent, which made the self-assembly ID8 naturally form polyelectrolyte–calcium complexes with β-sheet conformation. Overall, the self-assembly ID8 was a promising separate calcium medium to induce collagen mineralization [19]. As a peptide with only eight amino acid residues (≪40 kDa), ID8 monomer was easy to diffuse into collagen. If the calcium-responsive self-assembly of ID8 monomer inside collagen could be realized, these polyelectrolyte–calcium complexes located in the collagen were expected to give priority to nucleation inside collagen during mineralization. Hence, for the pretreatment in this study, collagen was first soaked in ID8 solution to make ID8 monomers enter the collagen by passive diffusion. Afterwards, the addition of calcium induced the self-assembly of ID8 inside and outside collagen. Finally, ID8 outside collagen was removed by DIW washing, and ID8 inside collagen remained. As the control, collagen in DIW group represented collagen pretreated with CaCl_2_ solution without ID8 pretreatment. Moreover, the ID8 monomer could enter and exit collagen freely based on its size, so in ID8 monomer group, only those molecules that were adsorbed onto collagen remained after being washed by DIW. This group reflected the effect of ID8 molecules that adsorbed onto collagen. The blue shift at amide A band indicated that the intermolecular hydrogen bonds were enhanced in self-assembly ID8 and ID8 monomer pretreated collagen (ATR-FTIR). XPS detection can reveal the subsurface atomic information at a depth of 2–5 nm. The calcium concentration of self-assembly ID8 pretreated collagen (1.45 at%) and ID8 monomer pretreated collagen (1.24 at%) was significantly higher than that of DIW group (0.72 at%), which can also be confirmed by the absorbance measurement of calcium. Moreover, the calcium peak of ID8 pretreated collagen was also significantly stronger under SEM-EDS. And the absorbance test of calcium directly proved the calcium retention ability of collagen gel in different groups. Similarly, the two groups pretreated with ID8 could retain more calcium than DIW group. The calcium retention may be due to the self-assembly of ID8 inside collagen, which was supposed to facilitate subsequent collagen mineralization. In addition, collagen gel pretreated with self-assembly ID8 showed minimal WCA than the other groups. These results suggested that self-assembly ID8 could significantly increase the hydrophilicity of collagen, which may also contribute to the promotion effect on collagen intrafibrillar mineralization. Interfacial wetting effect of biomolecules has been proved to be important in their regulation of biomineralization [34].

The mineralization experiment was first conducted on bare Type I collagen. Collagen of DIW group showed no intrafibrillar mineralization tendency due to the precipitation of crystals in mineralization medium at 1 and 3 days. Although intrafibrillar mineralization was not realized in PAA group at 1 day, the ACP infiltration indicated a tendency of intrafibrillar mineralization. As expected, intrafibrillar mineralization with highly ordered orientation appeared after 3 days of mineralization. Both self-assembly ID8 group and ID8 monomer group achieved fast intrafibrillar mineralization at 1 day. However, the mineralized collagen in self-assembly ID8 group still exhibited periodic structure, while that of PAA group and ID8 monomer group did not. What’s more, it was proved that self-assembly ID8 alone could not induce collagen intrafibrillar mineralization, PAA as the nucleating inhibitor was still necessary (Supplementary Fig. S1). The detailed mineralization process in ID8 group was further explored. Collagen intrafibrillar mineralization with lower crystallinity was almost achieved at 12 h. Interestingly, the intrafibrillar mineralization with improved crystallinity showed periodic structure again at 1 day, which was not visible in PAA group and ID8 monomer group. In general, Type I collagen is characterized by characteristic 67 nm periodic cross-striations or banding (D-periods), consisting of gap zones (40 nm) and overlap zones (27 nm). And in natural dentin or bone, HAP preferentially deposits in collagen gap zones leading to staggered nanostructures gives rise to a periodic banding pattern (67 nm), with the repeat motif corresponding to the D-periods. This kind of hierarchical intrafibrillar mineralized collagen endows dentin and bone with enough toughness to obtain anti-fracture ability [37, 41–44]. The effect of self-assembly ID8 pretreatment on fully demineralized dentin samples was also explored. Based on the above results, we believed that self-assembly ID8 was a better form of ID8 utilization, so the ID8 monomer group was not set in this part of experiments. Fully demineralization was confirmed by the combination of SEM, XRD and ATR-FTIR. In view of the complexity of dentin samples compared with bare collagen, this experiment was carried out for 3 and 7 days. Unfortunately, the efficacy of ID8 on dentin samples was much weaker than that of bare collagen, even if the differences among groups. Probably because all samples were rinsed in DIW with ultrasonic after mineralization, no occlusion of tubules was seen in all groups. As demonstrated by SEM, XRD combined with ATR-FTIR, all groups showed similar amorphous minerals deposition at 3 days. However, self-assembly ID8 group exhibited less extrafibrillar minerals at 7 days compared with DIW group and PAA group. It was speculated that only the samples’ surface had been mineralized due to the dense collagen layer of dentin and the crosslinking of various glycosaminoglycans, which may hinder the penetration of ACP. To confirm that, collagen layers on samples’ surface were cut for TEM detection [45]. As expected, only the self-assembly ID8 group showed hierarchical intrafibrillar mineralization.

The mechanism was also speculated, which may provide new insights into collagen mineralization. In the process of mineralization, ID8 inside collagen could attract nucleation precursor, calcium and phosphorus in mineralization medium through Coulombic attraction, then promoted the periodical penetration of ACP into collagen and finally induced hierarchical intrafibrillar mineralization [46, 47]. Moradian-Oldak *et al*. designed an amelogenin-inspired peptide P26, which could self-assemble into nanospheres through interaction with collagen and promote collagen intrafibrillar mineralization. One of the proposed mechanisms was that the small size of P26 monomers (<6 kDa) allowed P26 to readily diffuse into the water-filled compartments of collagen and then acted as the sequestration biomimetic analogue to recruit calcium ions actively and promote apatite nucleation. Similarly, ID8 adopts a smaller size than P26 and exhibits calcium responsive self-assembly, which naturally has the advantage of acting as a polyelectrolyte–calcium complex. Therefore, ID8 may promote collagen intrafibrillar mineralization in similar ways. In addition, different from the research of Liu *et al*., hierarchical collagen intrafibrillar mineralization was realized using high MW PAA (≫2 kDa) in our experiment. We speculated that ID8 may play an active role in guiding the preferential deposition of HAP into gap zones, rather than relying solely on the thermodynamic behavior differences between overlap zones and gap zones based on the structure of collagen. Another possible mechanism was that there may exist process of crystal dissolution and recrystallization [48], which was indicated by Fig. 7D–F, abundant minerals adsorbed on collagen surface after 6 h of mineralization, and some of them already began to crystallize. Part of these extrafibrillar minerals entered into collagen and part of them remained on the surface of collagen with the process of mineralization, which can be verified by Fig. 6Q and Fig. 6R. The orientation of *c*-axis of crystals was not along the fibril axis but nearly perpendicular to it in Fig. 6Q and Fig. 6R, indicating extrafibrillar minerals on collagen. However, these minerals almost disappeared with time. Either way, the calcium retention inside collagen may provide support for intrafibrillar mineralization. Collectively, intrafibrillar mineralization was promoted by the usage of the calcium-responsive self-assembly peptide ID8. However, such model was still insufficient in more complex fully demineralized dentin model, more effective pathway of ACP penetration into dentin may be explored in our further study.

Notably, compared with other biomolecules, the calcium-responsive self-assembly ability of ID8 endows it with the inherent advantage of forming polyelectrolyte–calcium complexes easily, and enables ID8 to perform regulatory effects similar to macromolecules in specific environments with a much smaller size. ID8 represents a class of peptides with self-assembly properties that can mimic the role of bioactive macromolecules through much smaller size. These types of peptides have financial and clinical advantages over bioactive macromolecules and are expected to be potential tools for biomimetic mineralization in the future.

## Supplementary data

Supplementary data are available at *REGBIO* online.

## Supplementary Material

rbac059_Supplementary_Data
