# Supplementary material for: Promoting effect of a calcium-responsive self-assembly β-sheet peptide on collagen intrafibrillar mineralization
Source: Regen Biomater. 2022 Sep 5;9:rbac059. doi: 10.1093/rb/rbac059 (PMC9514854; doi:10.1093/rb/rbac059)
Supplement: rbac059_Supplementary_Data [file rbac059_supplementary_data.docx]

**Promoting effect of a calcium-responsive self-assembly β-sheet peptide on collagen intrafibrillar mineralization**

Zhongcheng Li^1^, Qian Ren^1^, Sili Han^1^, Longjiang Ding^1^, Xi Qin^2^, Die Hu^1^, Ting He^1^, Tian Tian^1^, Ziqian Lu^1^, Linglin Zhang^1,*^

^1^State Key Laboratory of Oral Diseases, National Clinical Research Centre for Oral Diseases, Department of Cariology and Endodontics, West China Hospital of Stomatology, Sichuan University, Chengdu 610041, Sichuan, China; ^2^Department of Oral Medicine, Shenzhen Stomatology Hospital, Shenzhen 518038, Guangdong, China

**^*^**Correspondence address. State Key Laboratory of Oral Diseases, National Clinical Research Centre for Oral Diseases, Department of Cariology and Endodontics, West China Hospital of Stomatology, Sichuan University, No. 14, Section 3 of Renmin Road South, Chengdu, China. Tel: +86-28-85503470; Fax: +86-28-85581436; E-mail: zhll_sc@163.com


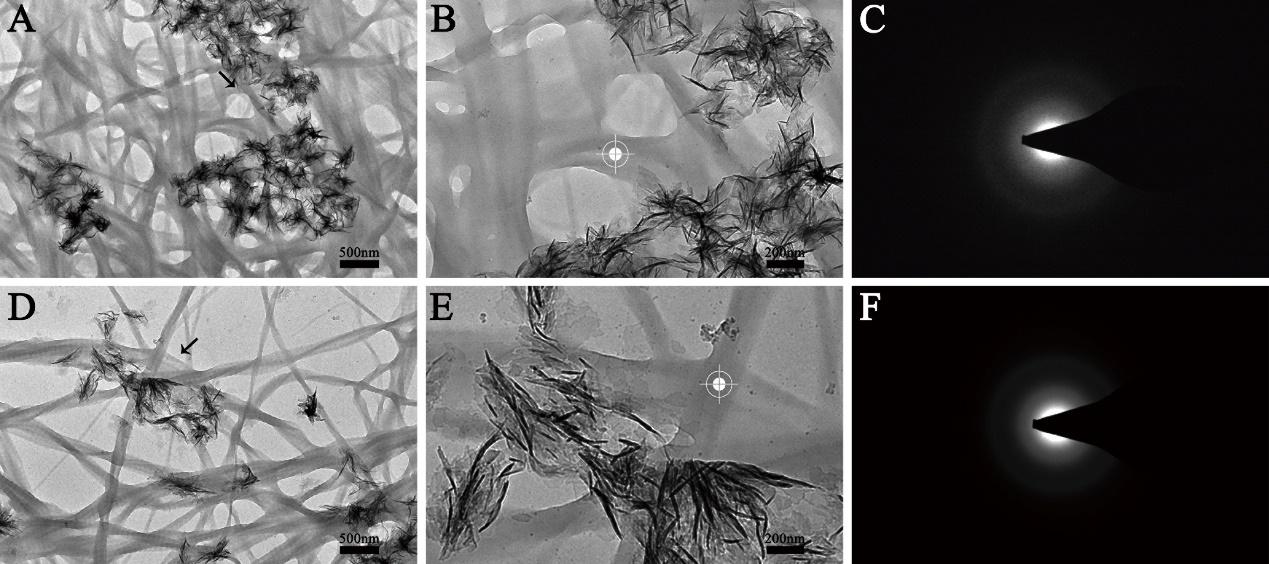


**Supplementary Figure S1**. TEM-SAED of the collagen pretreated with self-assembly ID8 and mineralized in medium without PAA for 1 d (**A-C**) and 3 d (**D-F**). (**B, E**) were corresponding zoom-in figures of the narrow black arrow marked areas in (**A, D**), and the corresponding SAED patterns were shown in (**C, F**). The white targets marked the diffraction areas.
